# Suppressed *SF3B1* Expression Lowers *METTL3* Transcription and m^6^A RNA Expression

**DOI:** 10.3390/ijms27125396

**Published:** 2026-06-15

**Authors:** Namjeong Choi, Hina Ashraf, Haihong Shen

**Affiliations:** 1 School of Life Sciences, Gwangju Institute of Science and Technology, Gwangju 61005, Republic of Korea; njchoi@ligachembio.com (N.C.);; 2 LigaChem Biosciences Inc., 10, Gukjegwahak 10-ro, Yuseong-gu, Daejeon 34002, Republic of Korea

**Keywords:** SF3B1, METTL3, transcription, m^6^A

## Abstract

Splicing factor 3b1 (SF3B1), a component of U2 small nuclear ribonucleoprotein (U2 snRNP), has been known for its essential roles in pre-mRNA splicing and alternative splicing. Here we show that knocking down (KD) of SF3B1 broadly induced a significant reduction in mRNA expression in the genome. One of the genes whose expression is reduced by SF3B1 KD is methyl-transferase-like 3 (*METTL3*), a writer of N6-methyladenosine (m^6^A). We demonstrate that expression of both *METTL3* mRNA and protein is affected by SF3B1 KD, which further decreases the m^6^A RNA expression level. m^6^A-seq indicates that SF3B1 KD affects m^6^A distribution within multiple genes in the genome. In addition, a high proportion of hypo-methylation events by SF3B1 KD (~70%) are overlapped in METTL3 KD cells, and a conserved m^6^A motif is observed in the hypo-methylated regions as in SF3B1 KD cells, suggesting the m^6^A decrease by SF3B1 is a direct effect of the reduced *METTL3* expression. Furthermore, RT-qPCR using unlabeled RNA and 5-Bromouridine (BrU)-labeled nascent RNA and actinomycin D treatment demonstrates that transcription of *METTL3* is significantly reduced but the mRNA decay rate is not altered, suggesting that *METTL3* expression is altered at the transcription level. We further show that SF3B1 interacts with RNA polymerase (Pol) II in the RNA independent manner, further indicating the involvement of SF3B1 in transcription. Lastly, we demonstrate that the transcription inactive H3K27me3 on the *METTL3* promoter was significantly increased whereas transcription active H3K4me3 was not changed by SF3B1 KD. Taken together, we conclude that reduced SF3B1 expression suppresses the transcription of *METTL3* and inhibits m^6^A RNA expression.

## 1. Introduction

Pre-mRNA splicing is a type of post-transcriptional process in which introns are removed and exons are ligated to produce mature RNA (mRNA). This process requires conserved sequences of 5′ splice sites (5′SS), 3′ splice sites (3′SS) and branch point sites (BPS) to occur. Among regulatory elements, the SF3B family complex containing splicing factor 3b subunit 1 (SF3B1) or SAP155 makes an essential part of U2 small nuclear ribonucleoproteins (snRNPs). SF3B1 is the largest and core component of U2snRNP complex and plays an important role in pre-mRNA splicing [1,2,3,4]. The interactions between SF3B1, U2AF65 and p14 are essential for branch point recognition by the spliceosomal complex (U2snRNP). For cleavage of pre-mRNA the base-pairing between U2 snRNP and BPS is an important event and this base-pairing is stabilized by SF3B1. During the formation and assembly of the pre-spliceosome complex, SF3B1 together with U2AF65 facilitates the recruitment of U2 snRNP to BPS [5,6]. Previous studies have shown that SF3B1 is the protein that makes direct contact with both sides of BPS in pre-mRNA [3,4]. It is also reported that SF3B1, together with p14, directly interacts with BPS adenosine [3]. In addition to single intron splicing, SF3B1 also regulates alternative splicing (AS) in the genome. One such example is alternative splicing of the *SMN* gene [1]. Among pre-mRNA splicing factors, SF3B1 shows frequent mutations in various cancers including chronic myelomenocytic leukemia (CMML), chronic lymphocytic leukemia (CLL), myelodysplastic syndrome (MDS), and breast and pancreatic cancers, and therefore it is the best target for cancer therapy [7,8,9,10,11]. For example, spliceostatin is a cancer drug that inhibits aberrant splicing of pre-mRNA by interfering with BPS selection of SF3B1 [12]. Mutated SF3B1 induces activation of cryptic 3′ splice sites and alternative usage of those BPS [13,14]. Mutated SF3B1 disrupts the contact between SURP and SUGP1, required for BPS selection that results in aberrant splicing [15].

SF3B1, in addition to its essential role in pre-mRNA splicing and alternative splicing, has also shown its potential role in gene expression as SF3B1 inhibitors sudemycin E and pladienolide B (PB) treatment induced changes in gene expression [16,17]. ChIP-seq analysis revealed that SF3B1 interacts with chromatin via RNA and this binding is specifically enriched at mono-nucleosomes near exon positions [18]. Another report showed that SF3B1 chromatin binding and its role in splicing are not dependent on each other [19]. Importantly, SF3B1 inhibitor sudemycin E treatment induces changes in chromatin modifications that subsequently result in changes in gene transcription [16]. Notably, SF3B1 also plays an important role in viral transcription by interacting with HIV Tat [20].

In eukaryotic cells, N6-methyladenosine (m^6^A) is the most abundant and conserved modification. Methyltransferase-like 3 (METTL3) is the most important writer of m^6^A modification [21,22,23,24]. The m^6^A modification pattern, revealed with the help of m^6^A-sequencing, shows that it is mostly distributed in 3′ untranslated regions (3′UTRs) near the stop codon and 5′ untranslated regions (5′UTRs) near the start codon, and is more abundant in long internal exons with the consensus sequence motif RRm^6^ACH [23,25,26]. During m^6^A modification METTL3 works with METTL14. METTL3 carries out the m^6^A deposition by acting as a catalytic subunit while METTL14 provides a scaffold for RNA binding [24,27,28]. Reversibly, m^6^A can be removed from mRNA by two m^6^A demethylases, FTO and ALKBH5 [29,30]. After m^6^A modification, reader proteins including the YT521-B homology (YTH) domain directly bind to the m^6^A modified mRNA and regulate many post-transcriptional modifications including mRNA decay, splicing, translation and polyadenylation [31,32,33]. In addition, m^6^A modification also regulates binding affinity of other RNA binding proteins like hnRNP C and hnRNP A2/B1, through regulating the secondary structure of the target mRNA [34,35,36]. Moreover, m^6^A modification also plays a role in various human diseases including initiation, progression and recurrence of tumor, obesity, neuronal disorders and immunological diseases [37,38,39,40,41,42]. m^6^A writers, readers and erasers can be used as a prognostic values in uveal melanoma [42].

Here we show that SF3B1 suppression broadly induced a significant reduction in mRNA expression in the genome. One of the genes that showed significant changes in expression by SF3B1 KD is *METTL3*. We demonstrated that expression of *METTL3* mRNA and subsequent protein expression is reduced by SF3B1 KD, which further decreased m^6^A modified RNA expression as shown with dot-blotting analysis. m^6^A-seq indicates that SF3B1 KD affects m^6^A distribution within multiple genes in the genome. In addition, a high proportion of hypo-methylation events by SF3B1 KD (~70%) is overlapped in METTL3 KD cells. In SF3B1 KD cells a conserved m^6^A motif is frequently observed in hypo-methylated regions, suggesting the m^6^A decrease by SF3B1 is a direct effect of the reduced *METTL3* expression. Furthermore, RT-qPCR using unlabeled RNA and 5-Bromouridine (BrU)-labeled nascent RNA and actinomycin D treatment demonstrated that synthesis of METTL3 is significantly reduced but the mRNA decay rate is not altered, further strengthening the notion that SF3B1 affects *METTL3* transcription. We further showed that SF3B1 interacts with RNA polymerase (Pol) II in the RNA independent manner, indicating that SF3B1 regulates *METTL3* transcription by interacting with RNA Pol II and thus is indirectly involved in regulating m^6^A deposition machinery. Lastly, we demonstrated that transcription inactive H3K27me3 on *METTL3* promoter was significantly increased whereas transcription active H3K4me3 was not changed by SF3B1 KD, showing that transcription inhibition of *METTL3* RNA occurs in SF3B1 KD. Taken together, we conclude that SF3B1 expression reduction suppresses transcription of *METTL3* and lessens m^6^A RNA expression which adds another regulatory layer in downstream mRNA processing.

## 2. Results

### 2.1. SF3B1 KD Reduced METTL3 Gene Expression

**Figure 1:**
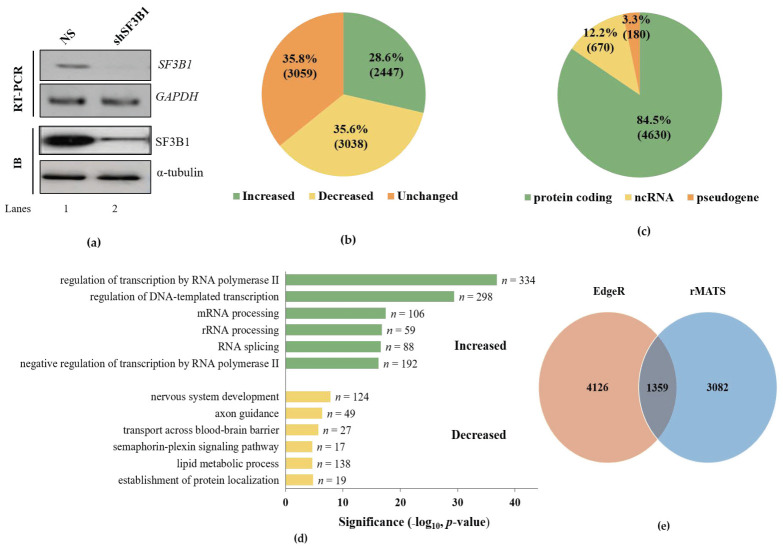
SF3B1 KD reduced *METTL3* gene expression: (**a**) (Upper 2 lanes) RT–PCR validation of SF3B1 KD in HEK293T cells and NS shRNA treated cells with GAPDH as loading control, (Lower 2 lanes) Immunoblotting analysis of SF3B1 protein in SF3B1 KD and NS shRNA treated cells with α–tubulin as loading control. (**b**) Pie chart illustrating the distribution of differentially expressed genes (DEGs) in SF3B1 KD compared to NS. DEGs were identified using EdgeR based on a significance threshold of *p*–value < 0.05 and|log_2_FC| ≥ 0.58 (1.5-fold change). (**c**) A pie chart showing classification of genes effected upon SF3B1 KD. (**d**) Gene ontology analysis of expression enhanced or reduced genes by SF3B1 KD (green bars represent upregulated gene number and their relevant biological process and yellow bars represent downregulated gene number and their biological function). (**e**) A pie chart representing overlapped genes showing changes in both expression as well as alternative splicing upon SF3B1 KD. All results were derived from three independent biological replicates (*n* = 3).

Previous reports demonstrated that the SF3B1 inhibitor sudemycin induces genome-wide changes in gene expression [16], implying a role of SF3B1 in gene expression. To directly investigate the roles of SF3B1 in gene expression, we applied RNA-sequencing from SF3B1 targeting shRNA (shSF3B1) and non-silencing shRNA (NS) treated HEK293T cells. Using this RNA-seq data, first we performed rMATs analysis to identify the transcriptome-wide effects of SF3B1 KD in alternative splicing [1], and then analyzed gene expression using the EdgeR tool [43]. Figure 1a shows the KD efficiency of SF3B1-targeting shRNA in HEK293T cells (SF3B1 KD and NS samples used for RNA-seq). As shown in Figure 1b, the expression of numerous genes in mRNA was increased (2447) or decreased (3038) upon SF3B1 KD while 3059 genes showed no change (Supplementary Table S2), most of which were protein-coding genes (84.5%) with small amounts of noncoding RNA (12.2%) and pseudogenes (3.3%) (Figure 1c) (Supplementary Table S3). Gene ontology enrichment analysis (using DAVID: https://davidbioinformatics.nih.gov/, accessed on 10 February 2024) [44], Ref. [45], showed that while genes upregulated upon SF3B1 KD were involved in regulation of transcription by RNA polymerase II, mRNA processing, rRNA processing, regulation of DNA template transcription and RNA splicing, downregulated genes were enriched in biological processes related to nervous system development, axon guidance, transport across blood–brain barrier, lipid metabolic processes and establishment of protein localization (Figure 1d) (Supplementary Table S4). We next asked whether this altered gene expression occurred by AS in SF3B1 KD. To address this question, we compared the genes of altered expression with the genes of altered AS (Supplementary Table S5). As shown in Figure 1e, 1359 genes overlapped in both groups. Notably, 4126 genes were regulated only at the gene expression level, which was not observed in altered splicing events (SE, MXE, IR, A5SS, and A3SS), indicating that in addition to AS, other aspects of gene expression are also affected/regulated by SF3B1 (U2snRNP core splicing component).

**Figure 2:**
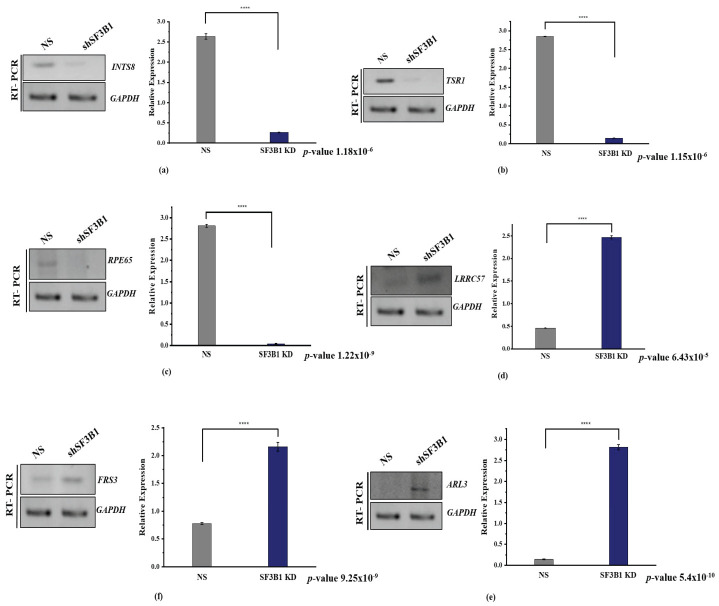
RT-PCR validation of differential gene expression results: (**a**–**f**) RT-PCR validation of *INTS8*, *TSR1*, *RPE65*, *LRRC57*, *ARL3* and *FRS3* genes in *SF3B1* KD vs. NS cells with *GAPDH* as loading control. Band intensities were quantified using ImageJ v1.53e and normalized to GAPDH. SEM from three independent experiments is illustrated using error bars. Statistical significance is shown as **** *p* < 0.0001.

To validate the gene expression changes (EdgeR analysis) in vitro, we performed RT-PCR experiments for three enhanced and three reduced expression genes for SF3B1 KD and NS samples. IGV plots showed that expression peaks of *INTS8* (Integrator complex subunit 8 ENSG00000164941), *TSR1* (Ribosome maturation factor ENSG00000167721) and *RPE65* (Retinoid isomerphydrolase ENSG00000116745) were decreased (Supplementary Figure S1). Consistent with RNA-seq results, RT-PCR results demonstrate that whereas expressions of *LRRC57*, *FRS3* and *ARL3* RNA were increased, whose log_2_FC values indicated 5, 3.7 and 3-fold increases in expression levels respectively, RNA expressions of *INTS8*, *TSR1* and *RPE65* were decreased, whose log_2_FC values indicated 4, 4 and 8-fold decreases in expression levels respectively (Figure 2a–f). These results confirm that SF3B1 regulates gene expression in the genome.

**Figure 3:**
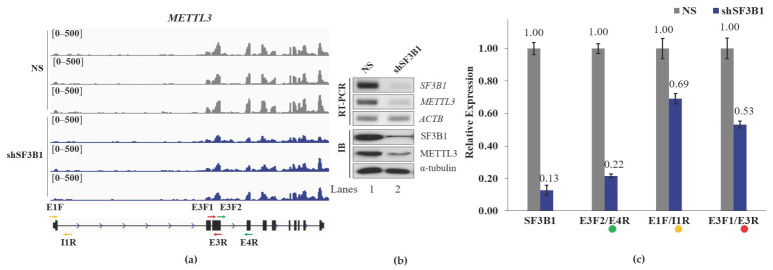
*METTL3* transcript expression was reduced by SF3B1 KD: (**a**) IGV showing that expression peaks of *METTL3* RNA was decreased by *SF3B1* KD; (**b**) (upper 2 lanes) RT-PCR validation of *SF3B1* KD in HEK293T cells and NS shRNA treated cells showing *SF3B1* KD reduced *METTL3* expression with *ACTB* as loading control, (lower 2 lanes) immunoblotting analysis of SF3B1 and METTL3 proteins in SF3B1 KD and NS shRNA treated cells with α-tubulin as loading control. (**c**) RT-qPCR analysis of *METTL3* mRNA in SF3B1 KD cells and NS shRNA treated cells. Primers used for RT-qPCR are indicated with arrows at bottom of (**a**). Green dot/arrow represents forward primer binding position in exon 3 and reverse primer binding position in exon 4, yellow dot/arrow represents forward primer binding position in exon 1 and reverse primer binding position in intron 1 and red dot/arrow indicates forward primer binding position at start of exon 3 and reverse primer binding position at ending of exon 3 of *METTL3* gene in RT-qPCR experiments. Mean ± SD is represented from three independent experiments using error bars.

We noticed that the expression of *METTL3*, a writer of m^6^A mRNA modification, was decreased in RNA-seq data of SF3B1 KD, so we selected this gene among genes with decreased expression upon SF3B1 KD (Supplementary Table S2). Integrative Genome Viewer (IGV) showed that *METTL3* RNA was reduced significantly in triplicated SF3B1 KD cells compared with control cell RNA-seq (Figure 3a). We first performed RT-PCR and immunoblotting analysis to demonstrate that both *METTL3* RNA and protein levels were decreased by SF3B1 KD (Figure 3b) indicating that SF3B1 KD directly suppressed *METTL3* gene expression. We further performed more accurate RT-qPCR of *METTL3* RNA. As shown in Figure 3c, *METTL3* RNA expression was significantly reduced in SF3B1 KD cells in RT-qPCR experiments with different primer sets (E3F2/E4R, E3F1/E3R, and E1F/I1R) (Figure 3c RT-qPCR; Figure 3a lower panel showing primer binding positions). Thus, SF3B1 possibly regulates transcription of the *METTL3* gene.

### 2.2. SF3B1 Reduced m^6^A Level of mRNA

**Figure 4:**
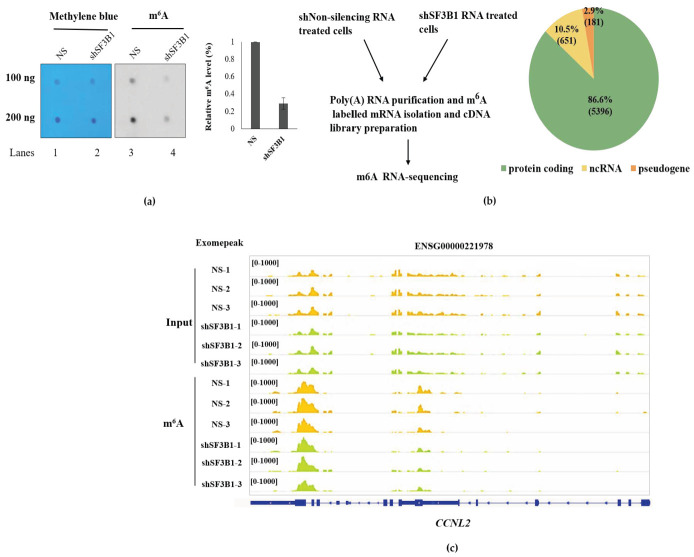
SF3B1 reduced m^6^A level of mRNA: (**a**) SF3B1 KD and NS shRNA treated cells. Methylene blue assay was used as a control. (Right) Quantitative analysis of m^6^A RNA in SF3B1 KD and NS shRNA treated cells (200 ng); (**b**) (**Left**) strategy of m^6^A sequencing in SF3B1 KD cells, (**Right**) a pie chart showing genes with reduced m^6^A expression by SF3B1 KD; (**c**) IGV showing that m^6^A peaks of *CCNL2* were decreased by SF3B1 KD. Input RNA-seq results of *CCNL2* are shown as controls.

*METTL3* is the catalytic subunit of the m^6^A RNA writer [24]. We then asked whether reduced *METTL3* mRNA/protein expression caused by SF3B1 also induces decreased m^6^A RNA expression in mRNA. To confirm this, we performed dot-blotting analysis with the anti-m^6^A antibody for mRNAs from SF3B1 KD and non-silencing (NS) shRNA treated cells, and methylene blue analysis was used as a control. As expected, whereas total mRNA levels were similar in the methylene blue assay (lanes 1–2), m^6^A RNAs in SF3B1 KD cells were reduced significantly (~30%) compared to NS shRNA treated cells (lanes 3–4) (Figure 4a). Thus, SF3B1 KD induces a decrease in m^6^A RNA expression.

To investigate whether SF3B1 broadly affects the m^6^A level in the transcriptome, we performed N6-methyadenosine-sequencing (m^6^A-seq) with poly(A) RNAs obtained from SF3B1 KD and non-silencing shRNA treated cells (Figure 4b, left), followed by differential methylation analysis using the exomePeak tool v2.16.0 (a software to identify enriched binding/methylation sites for short read sequencing data) [46]. As we expected, we could observe hypo-methylation sites within 6228 genes in SF3B1 KD cells out of which 5396 are protein-coding genes (Figure 4b), suggesting that reduced *METTL3* expression by SF3B1 KD induced decreased m^6^A distribution. We further analyzed the gene identity of hypo-methylated genes by SF3B1 KD; as shown in Figure 4b, protein-coding genes were predominant (86.6%), whereas non-coding (10.5%), and pseudogenes (2.9%) were also observed (Supplementary Table S6). Among the hypo-methylated sites in SF3B1 KD cells, we selected *CCNL2* to demonstrate the reduction in m^6^A peaks in SF3B1 KD cells, whereas its total RNA level was not altered (Figure 4c).

**Figure 5:**
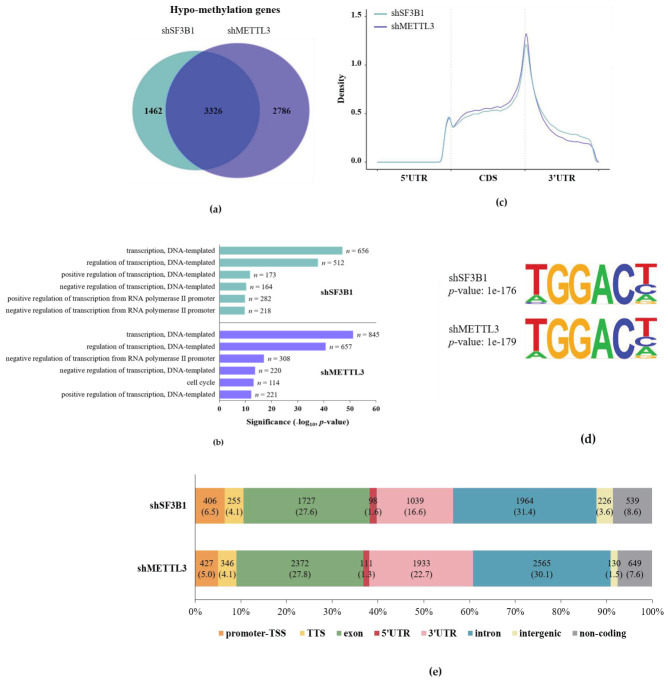
Comparing m^6^A distribution in SF3B1 and METTL3 KD: (**a**) Overlapped hypo-methylated genes in SF3B1 and METTL3 KD cells; (**b**) GO analysis (DAVID) of hypo-methylated mRNAs in SF3B1 and METTL3 KD cells; (**c**) Metagene plot for the position of m^6^A peaks in SF3B1 and METTL3 KD cells; (**d**) HOMER motif enrichment analysis of hypo-methylated positions in SF3B1 and METTL3 KD cells; (**e**) Stacked horizontal bar chart for m^6^A peak annotations. Peak annotations are composed of promoter–TSS (by default defined from −1 kb to + 100 bp), TTS (by default defined from −100 bp to +1 kp), exon, 5′UTR, 3′UTR, intron, intergenic and non-coding region.

To ask whether hypo-methylation of RNA by SF3B1 KD occurs in a similar way to that by METTL3, we also performed m^6^A-seq with RNAs from METTL3 KD cells. Consistent with the previous reports [24], we found 6112 hypo-methylation sites by METTL3 KD (Figure 5a). We further compared the significantly hypo-methylated genes detected in SF3B1 KD with that in METTL3 KD cells (4788 and 6112, respectively) (Supplementary Tables S7 and S8 showing all hyper- and hypo-methylated genes). As shown in Figure 5a, ~70% of the hypo-methylated genes (3326 in 4788) by SF3B1 KD were also found in the list of METTL3 KD genes, indicating that SF3B1 KD has a similar impact on m^6^A distribution as the direct METTL3 KD. Gene ontology analysis shows that hypo-methylated genes by SF3B1 KD are involved in many biologically important processes like transcription by RNA polymerase and DNA-templated transcription (Figure 5b) (Supplementary Table S9), implying the potential roles of SF3B1 in regulating transcription as well as downstream RNA processing.

We next wondered whether the altered m^6^A peak distribution by SF3B1 KD has similar trends to that by METTL3 KD. As expected, hypo-methylation sites by SF3B1 KD were highly enriched in 3′UTRs near the stop codon, and 5′UTRs near the start codon, with a similar distribution to METTL3 KD, highly consistent with previous reports [23,25] (Figure 5c). We next applied the HOMER motif analysis tool (a novel regulatory elements motif discovery algorithm) [47] to exomePeak results to find hypo-methylated motifs by SF3B1 KD. As expected, similar to the control METTL3 KD cells, a subset of the m^6^A motif GGAC is highly enriched in the hypo-methylation sites by SF3B1 KD, highly matching the m^6^A consensus sequence (Figure 5d). We further compared the annotated regions in the genome where m^6^A peak decreases appeared by SF3B1 KD or METTL3 KD. We found that these two groups demonstrated quite similar annotations of m^6^A peaks with some differences; for example, there were more promoter-transcription start sites (TSS, from −1 kb to +100 bp) and fewer 3′UTR m^6^A peaks in SF3B1 KD than METTL3 KD cells (Figure 5e). In conclusion, the results above indicate that decreased *METTL3* expression by SF3B1 KD widely affects m^6^A RNA distributions in the transcriptome.

### 2.3. SF3B1 KD Affects Transcription but Not mRNA Decay of METTL3 Gene

**Figure 6:**
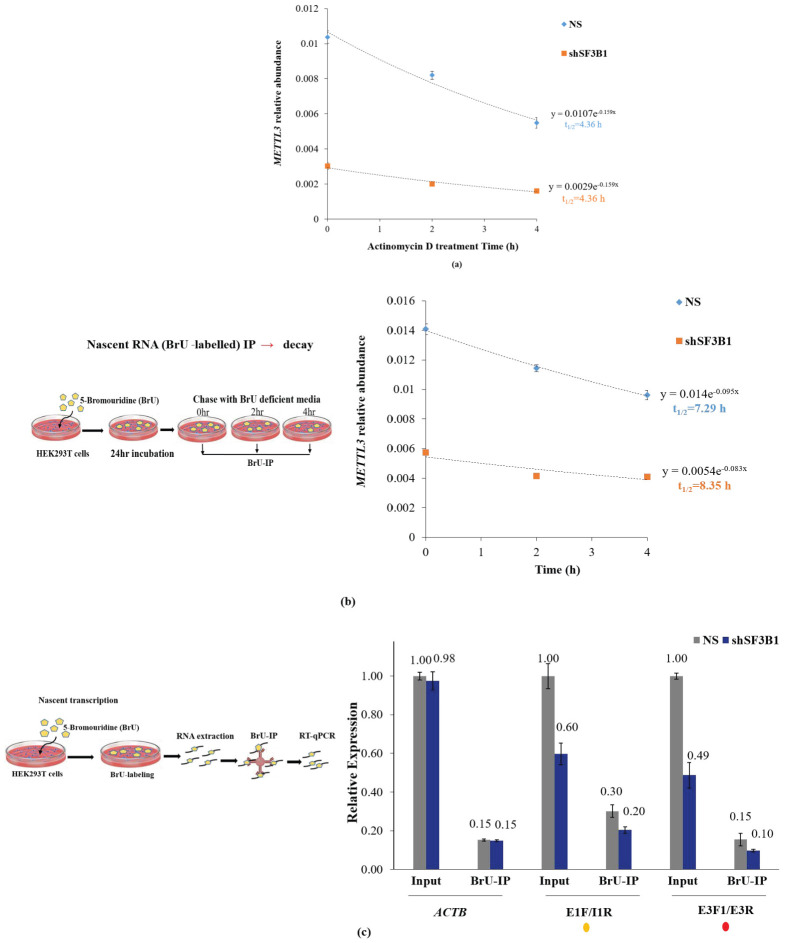
SF3B1 KD affects transcription but not mRNA decay of *METTL3* gene: (**a**) Remaining *METTL3* mRNAs at 0 h, 2 h and 4 h following ActD treatment in SF3B1 KD and NS shRNA treated cells are shown. Formulas and half-lives were calculated on the relative amount of expression. (**b**) Left panel shows schematic diagram of BrU–chase experiment while right panel presents RT-qPCR results of BrU pulse-labeling for 24 h and chasing with BrU-deficient media for 0, 2 and 4 h. BrU-labeled RNA was isolated through BrU antibody and used for RT-qPCR. (**c**) SF3B1 KD and NS cells were treated with 2 mM BrU for 3 h, BrU-labeled RNA was isolated and RT-qPCR was performed. Data are presented as mean ± SEM from three independent experiments (*n* = 3). Transcript half-lives ($t_{1/2}$) were determined by fitting the data to a non-linear one-phase exponential decay model ($y=Ae^{-kx}$). All regression curves showed a goodness of fit with *R*^2^ > 0.8.

There are two possibilities for the reduced *METTL3* mRNA expression by SF3B1 KD, one of which is an increased *METTL3* mRNA decay by SF3B1 KD, and the other one is a decrease in *METTL3* mRNA transcription. To address the problems, we performed the mRNA decay assay by treating cells with actinomycin D, an inhibitor of RNA polymerase in eukaryotes, followed by RT-qPCR every two hours for four hours to calculate the half-life of the *METTL3* transcript. At the zero-time point, *METTL3* mRNA of SF3B1 KD cells was significantly lower than NS-shRNA treated cells, indicating that lentivirus targeting-SF3B1 already results in the reduction in the *METTL3* transcript (Figure 6a). Notably, we observed that the half-life of *METTL3* mRNA in SF3B1 KD cells (t_1/2_ = 4.36 h) was the same as NS treated cells (t_1/2_ = 4.36 h). To further validate the above results, we performed the mRNA decay assay of *METTL3* mRNA with nascently synthesized mRNA. To this end, we added 5-Bromouridine (BrU), a uridine analog with low cytotoxic effect on cells, into culture medium for 24 h, and then chased with BrU-deficient media. Immunoprecipitation (IP) assay using anti-BrU antibody and qRT-PCR analysis of *METTL3* mRNA every 2 h for 4 h was used to compare the stability of *METTL3* mRNA (Figure 6b, left). Consistent with previous *METTL3* mRNA decay results (Figure 6a), BrU labeled nascent *METTL3* mRNA was significantly reduced in SF3B1 KD cells compared to control cells (zero-time point) (Figure 6b, right). Furthermore, the half-life of SF3B1 KD cells (t_1/2_ = 8.35 h) was higher than NS cells showing that in SF3B1 KD cells the *METTL3* mRNA decay was slower than NS cells (t_1/2_ = 7.29 h) further ruling out the possibility of decay as the cause of low *METTL3* transcript in SF3B1 KD cells (Figure 6b, right). Although there is a modest difference (Figure 6b) in the half-lives of SF3B1 KD and NS samples, the overall decay kinetics were not substantially altered suggesting transcriptional suppression as the underlying mechanism for reduced *METTL3* expression. Therefore, these results in Figure 6a,b demonstrate that SF3B1 KD did not affect *METTL3* mRNA decay. Based on the fact that *METTL3* mRNA was significantly reduced in SF3B1 KD cells before adding the transcription inhibitor (actinomycin D assay zero-time point, Figure 6a right panel), we wondered whether SF3B1 KD affects the transcription of *METTL3* mRNA. To support this point, we compared the synthesis of nascent *METTL3* mRNA levels in SF3B1 KD and control cells using BrU labeled RNA followed by RT-qPCR analysis (Figure 6c). As shown in Figure 6c, whereas total input RNA and total nascent RNA remained at a similar level in SF3B1 KD and control cells, a significant decrease in nascent *METTL3* mRNA was observed in SF3B1 KD cells (~46%). Combined together, we conclude that silencing of SF3B1 affects RNA transcription but not RNA decay of *METTL3*.

### 2.4. SF3B1 Interacts with the RNA Pol II and Affects Its Occupancy on the Genome

**Figure 7:**
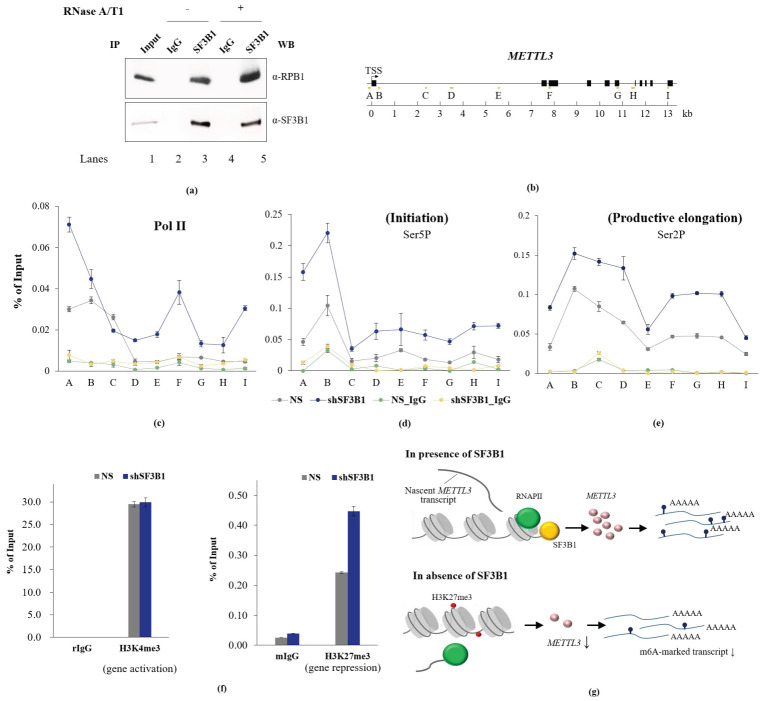
SF3B1 interacts with the RNA Pol II and affects its occupancy on the genome: (**a**) CoIP analysis of SF3B1 and RNA Pol II in the presence or absence of RNase A1/T. IP with anti-SF3B1 antibody was followed by IP with anti-RPB1 antibody. IP with anti-IgG antibody is shown as a control. (**b**) Schematic diagram of *METTL3* gene (13 kb). TSS is indicated by a vertical curved arrow. Primers in ChIP-qPCR are shown with a yellow line. (**c**–**e**) ChIP-qPCR analysis using RNA Pol II, RNA Pol II-Ser5P, and RNA Pol II-Ser2P antibodies and primers shown in (**b**) in SF3B1 KD and NS shRNA treated cells. (**f**) ChIP assay with H3K4me3 or H3K27 antibody followed by RT-qPCR in *METTL3* promoter in SF3B1 KD and NS shRNA treated cells. ChIP with anti-IgG antibody is shown as controls. Changes in chromatin modification upon SF3B1 KD showing an increase in H3K27me3 distribution resulting in *METTL3* transcription repression in SF3B1 KD cells vs. NS cells. (**g**) Proposed schematic diagram showing effects of involvement of SF3B1 in *METTL3* transcription.

Previous reports demonstrate that SF3B1 is associated with chromatin [18,19], and our results above showed that SF3B1 KD decreased *METTL3* transcription. Thus, we considered the possibility that SF3B1 interacts with RNA Pol II, the main player in eukaryotic transcription. To address this point, we performed co-immunoprecipitation (coIP) experiments in which IP with anti-SF3B1 antibody was followed by immunoblotting with anti-α-RPB1, the catalytic and largest subunit of RNA polymerase II. Figure 7a shows that SF3B1 interacted with α-RPB1 (lane 3), suggesting the interaction between SF3B1 and RNA Pol II. Surprisingly, RNase A/T1 treatment enhanced the interaction between SF3B1 α-RPB1 (Lanes 3 and 5), indicating that the interaction of SF3B1 with RNA Pol II is not dependent on the presence of RNA, and that this interaction is interfered with by the presence of RNA. We next analyzed RNA polymerase II occupancy on the *METTL3* genome, which comprises 13.2 kilobases (kb) of DNA, by using chromatin immunoprecipitation (ChIP) analysis with anti-α-RPB1 antibody followed by qPCR specific to different regions in its genome (A–I) (Figure 7b). In non-silencing cells, RNA Pol II was distributed across both the promoter and gene body in the *METTL3* gene, indicating efficient and processive transcription (Figure 7c). However, in SF3B1 KD cells, increased occupancy of Pol II on the promoter and gene body occurred, suggesting promoter pausing of Pol II and that decreased elongation occurred in SF3B1 KD cells. To validate these results, we applied two other forms of antibodies to RNA Pol II: the first one is against phosphorylated Ser5 (Ser5P) in the C-terminal domain (CTD) of Pol II that occurs during the transcription initiation stage, and the second one is against the phosphorylated Ser2 that occurs during productive elongation [48,49]. As expected, we found that occupancy of the initiating Ser5P was greatly increased near the TSS (Figure 7d), supporting that promoter pausing was increased by SF3B1 KD. In addition, elongating Ser2P was more enriched along the gene body of *METTL3* (Figure 7e), suggesting that transcription elongation was slowed down by SF3B1 KD. These results above demonstrate that SF3B1 interacts with RNA polymerase II and affects its occupancy on the *METTL3* genome.

The transcription change in *METTL3* by SF3B1 KD suggests an altered chromatin modification. It has been reported that U2 snRNPs are associated with histone H3 lysine 27 trimetylation (H3K27me3) [50]. We then tested the influence of SF3B1 KD on histone modifications on the *METTL3* promoter. We performed IP with antibodies against either histone H3 lysine 4 trimetylation (H3K4me3) involved in active transcription or H3K27me3 involved in inactive transcription, and then qRT-PCR targeting for the *METTL3* promoter region was followed. Figure 7f shows that H3K4me3 distribution on the *METTL3* promoter was not affected by SF3B1 KD. By contrast, H3K27me3 distribution was significantly increased in SF3B1 KD cells compared to NS shRNA treated cells. Therefore, under normal physiological conditions (in the presence of SF3B1) *METTL3* transcription is increased (increase in active chromatin modification) resulting in more m^6^A deposition on mRNA and SF3B1 KD increased transcription inactive chromatin modification leading to less *METTL3* transcript production and subsequently less m^6^A deposition on mRNA (Figure 7g).

## 3. Discussion

U2 snRNP is an important multi-protein part of spliceosomal machinery and SF3B1 is an essential component of this multiprotein complex as it regulates and stabilizes the binding between U2snRNP and pre-mRNA. The importance of proper functioning of SF3B1 can be understood from the fact that it is most frequently mutated in many cancers causing aberrant splicing by activating cryptic splice sites. In this report, we have studied the role of SF3B1 in gene expression in greater detail. Our RNA-seq analysis shows that both enhanced and reduced expressions of many functionally important genes are observed by SF3B1 KD. Furthermore, these genes are not overlapped with AS regulated genes by SF3B1 silencing indicating that SF3B1 has an essential role not only in AS but in gene expression as well. In addition, a few reports indicate its functions in the mRNA export, mRNA decay, and transcription [20,51,52]. In addition, Spliceostatin A (SSA), known to affect BPS recognition by SF3B1, causes gene expression changes, premature cleavage, and polyadenylation in addition to aberrant splicing [16,53]. Indeed, splicing factors have been shown to be involved in multiple functions in gene expression other than splicing [54,55,56,57]. Consistently, our results indicate that SF3B1 regulates many other aspects of gene expression in addition to its well-known functions in RNA splicing. But these other roles of SF3B1 in transcription, mRNA export and mRNA decay still need to be studied in greater detail.

In this paper we focused on *METTL3*, a well-known writer of m^6^A. Importantly, we have identified *METTL3* gene expression as a target of SF3B1 (Figure 7g). We have shown that SF3B1 regulates m^6^A RNA expression through regulating *METTL3* expression. SF3B1 KD causes hypo-methylation of m^6^A in thousands of genes, and most of them are overlapped with genes affected by *METTL3* KD. Interestingly, gene ontology analysis demonstrates that transcription-related functions are enriched in the hypo-methylated genes. In addition, we found that hypo-methylation regions due to SF3B1 KD are consistent with the m^6^A consensus motif, indicating its functions in m^6^A deposition through regulating *METTL3* expression. m^6^A is the most prevalent and essential modification controlling many aspects of mRNA regulation including transcription, splicing, polyadenylation, translation, mRNA export, and its distributions affecting human diseases [31,38,40,58,59]. Thus, gene expression changes by SF3B1 KD might also be caused by altered m^6^A distributions, which affect transcription and post-transcriptional handling of pre-mRNA.

Furthermore, the roles of SF3B1 in various diseases should also be studied through m^6^A-oriented gene expression. Frequent m^6^A-oriented functional pathway mutations of SF3B1 might provide important insight into cancer research. We also observed that some hypo-methylations by SF3B1 were not overlapped with that by *METTL3*, implying that another pathway of SF3B1 regulation in m^6^A distributions also exists.

Our ChIP-seq analysis showed that although the occupancy of RNAPII increases on the *METTL3* promotor and gene body, this increase does not lead to more transcription/expression of *METTL3*; rather, it happens as a result of promoter pausing and stalled elongation of RNAPII. Both Ser5 and Ser2 phosphorylated forms of RNAPII showed increased distribution in SF3B1 KD cells indicating that not only is promoter clearance delayed but elongation is also slowed down in SF3B1 KD cells. This indicates that the change in *METTL3* mRNA expression by SF3B1 KD is due to the alteration in *METTL3* RNA synthesis but not RNA decay. These results are consistent with previous reports that SSA regulates Pol II transcription [60]. Accordingly, we also observed the interaction between SF3B1 and RNA Pol II independent of the presence of RNA. This shows that SF3B1 is associated with nucleosomes that affect transcriptional and splicing outcomes irrespective of the presence of RNA [18,19]. The fact that this association between SF3B1 and RNAPII regulates *METTL3* expression which then regulates m6A deposition and downstream processing of mRNA reveals that SF3B1 may have a potential role in m^6^A machinery. This result is further strengthened by changes in histone modifications that are linked to transcription. We found that SF3B1 KD induced the increase in transcription inactive H3K27me3 on the *METTL3* promoter, but not active H3K4me3, suggesting roles of SF3B1 in transcriptional regulation of m^6^A machinery.

Although the interactions of SF3B1 with RNAPII have already been reported, our study established a link between SF3B1 and m^6^A regulation at the transcriptional level through *METTL3* transcription. This link suggests that transcription and downstream processing of mRNA through the involvement of SF3B1, in addition to AS, is regulated by some unknown molecular pathway that still needs to be investigated. Additionally, the fact that some hypo-methylated events due to SF3B1 KD did not overlap with *METTL3* KD hypo-methylated events suggests that there may be other SF3B1-dependent m^6^A regulatory pathways that warrant further study. Moreover, considering the fact that SF3B1 is most frequently mutated in many human cancers, the effects of SF3B1 mutations on m^6^A deposition may open new avenues for disease interventions.

## 4. Materials and Methods

### 4.1. Cell Culture, Transfection, shRNA Treatment, and Actinomycin D Treatment

Dulbecco’s Modified Eagle’s Medium (DMEM) supplemented with 10% FBS was used to grow HEK293T cultures (NCCC, USA). Polyethylenimine (PEI) (Sigma, St. Louis, MO, USA) reagent was used to facilitate the transfection of plasmids into cells. Cells were incubated for 2 days’ post transfection and TRI reagent was used to extract total RNA for RT-PCR validation. shRNA containing viruses were produced using HEK293T cell cultures using viral packaging and envelope plasmids (psPAX2 and PMD2G) together with target shRNA plasmids (Open Biosystems, Huntsville, AL, USA) in the presence of PEI to facilitate plasmid entry into cells. Viral supernatant was collected 48 h post transfection and filtered through 0.45 μm filter to remove cell debris. HEK293T cells were infected with shRNA viruses along with 5 mg/mL polybrene (Sigma, St. Louis, MO, USA) treatment. Total RNA was isolated 72 h’s post-infection for RT-PCR validation. Actinomycin D (10 μg/mL) was used to inhibit transcription in HEK293T cells 72 h post-infection with shRNA virus treatment. After transcription inhibition treatment RNA was extracted at different time intervals of 0, 2, and 4 h and RT-qPCR was performed to measure remaining transcripts using β-actin (ACTB) as a control [1].

### 4.2. RNA Extraction, RT-PCR and RT-qPCR

For total RNA extraction TRI reagent^®^ (TR118; Molecular Research Center, Cincinnati, OH, USA) was used according to manufacturer’s instructions. For reverse transcription, 1 μg of total RNA was used to make cDNA in the presence of moloney murine leukemia virus (M-MLV) reverse transcriptase (Elpis) and oligo-dT18 primer. cDNA (1 µL) was then used to perform PCR using target genes-specific primers. PCR products were then run on 2% agarose gels and stained with ethidium bromide (EtBr) for visualization. KAPA SYBR FAST kit (KK4606; KAPABIOSYSTEMS, Boston, MA, USA) was used to perform quantitative RT-PCR (RT-qPCR) following manufacturer’s instructions with ACTB as control [61].

### 4.3. mRNA Purification and m^6^A Dot Blot Analysis

Two step purification using Oligo d(T)25 Magnetic Beads (1419S; NEB, Ipswich, MA, USA) was used to purify mRNA. After purification and dilution, RNA denaturation was carried out at 95 °C heat block for 3 min. RNA was then directly blotted onto Biodyne B Membrane (60207; Pall Laboratory, Jasper, AL, USA) and air dried. Stratagene UV Stratalinker 2400 (Agilent Genomics, Santa Clara, CA, USA) was used to crosslink mRNA and membrane with two rounds of UV crosslinking. After crosslinking, 5% skim milk in 1X TBST was used for membrane blocking. Membranes were then incubated with m^6^A antibody (Synaptic Systems, 202-003) overnight. Methylene blue solution (0.02% methylene blue (*w*/*v*) in 0.3 M sodium acetate (pH 5.5)) was used for control blotting [62].

### 4.4. Methylated (m^6^A) RNA Immunoprecipitation (MeRIP)-Seq

EpiMark^®^ N6-Methyladenosine Enrichment Kit (E1610S; New England Biolabs, Ipswich, MA, USA) was used to perform MeRIP-Seq. Dynabeads™ Protein G (10004D; Invitrogen, Carlsbad, CA, USA) 25 μL and N6-Methyladenosine Antibody 1 μL were incubated on 4 °C rotator for 2 h. Fragmentation buffer (100 mM Tris (pH 8.0), 8 mM MgCl_2_) was then used to fragment purified RNA at 95 °C for 10 min with spike-in control RNA (m^6^A and unmodified, 0.1 fmol of each RNA). After, fragmentation RNA was concentrated using RNA Clean & Concentrator™-5 kit (R1014; ZYMO RESEARCH, Irvine, CA, USA) and 5% was saved as input. The remaining fragmented RNA (95%) was incubated with beads/antibody complexes. Buffer RLT (79216; QIAGEN, Hilden, Germany) was used for RNA elution, and precipitated following ethanol precipitation. Precipitated RNA was then used for library construction [63].

### 4.5. Sequencing Data Analysis

All sequencing experiments were performed in triplicate using the Illumina NovaSeq platform (Annoroad Gene Technology, Beijing, China) with a 150 bp paired-end (PE150) strategy. Raw data quality was assessed with FastQC and adapter trimming was conducted using Trim Galore. Then, processed reads were subsequently aligned to the human reference genome (hg38) using HISAT2.

For epitranscriptomic analysis, exomePeak v2.16.0 [46] (https://bio.tools/exomepeak accessed on 1 March 2024) and HOMER v 4.11 [47] were employed to identify differential m^6^A methylation peaks, enriched motifs, and gene annotation. To evaluate transcriptomic variations, differential gene expression analysis was performed using the EdgeR v4.1.0, while alternative splicing events were characterized using rMATS v3.28.0.

### 4.6. 5-Bromouridine (BrU) Immunoprecipitation (IP) and BrU IP Chase (BRIC)-qPCR

HEK293T cells 72 h post-infection were labeled with BrU (850187; Sigma-Aldrich, St. Louis, MO, USA) 2 mM for 3 h for BrU-IP. Cells were treated with DNase I (E1009-A; ZYMO RESEARCH, Irvine, CA, USA) and total RNA was extracted after DNase I treatment. RNA was precipitated with phenol–chloroform extraction and ethanol precipitation. Dynabeads™ Protein G beads (20 μL) were suspended in RSB-100 buffer (10 mM Tris-Cl (pH 7.5), 100 mM NaCl, 2.5 mM MgCl2, 0.4% Triton X-100). Anti-BrdU antibody (Sigma, B2531) 2 μL and tRNA 2.5 μg were added to beads and incubated for 1 h at 4 °C with constant rotation. Purified RNA 10 μg, tRNA 2.5 μg and RNase Inhibitor Murine (M0314S; NEB, Ipswich, MA, USA) were added to the beads respectively and further incubated 1 h. BrU-labeled RNA was then eluted from beads by adding buffer RLT (250 μL) and tRNA 2.5 μg and placing it at 80 °C heat block for 10 min. RNA Clean & Concentrator™-5 was used to purify eluted RNA. RT-qPCR was performed to quantify newly synthesized transcript. For BRIC, 48 h post shRNA treatment, BrU-labeling was performed for 24 h with 2 mM BrU. After labeling, cells were incubated with BrU-deficient media, and RNA was extracted at different time intervals of 0, 2, and 4 h, and the same process was followed for BrU-IP [64]. Beta-actin was used as an internal reference for normalization. Although endogenous controls can be subject to degradation during transcriptional inhibition, beta-actin exhibited consistent stability trends across both control and knockdown conditions, providing a reliable baseline for comparing the relative decay kinetics of target mRNAs.

### 4.7. Co-Immunoprecipitation (Co-IP)

SureBeads™ Protein A Magnetic Beads (1614013; Bio-Rad Laboratories, Hercules, CA, USA) were washed and incubated with NET-2 buffer along with anti-SF3B1 antibody (A300-996A; Bethyl Laboratories, Montgomery, TX, USA) overnight at 4 °C with constant rotation. HEK293T cells were suspended in NET-2 buffer (50 mM Tris-Cl (pH 7.5), 150 mM NaCl, 0.05% NP-40, protease inhibitor cocktails) and sonicated for 10 min. Lysate was treated with RNase A/T1 Mix (EN0551; Thermo Fisher Scientific, Vilnius, Lithuania) at RT rotator for 20 min. Beads/antibody complexes were incubated with pre-cleared lysate and incubated for 3 h at 4 °C with constant rotation and washed with NET-2 buffer to remove unbound RNA. Beads were mixed with 5X SDS loading dye and run on 6% SDS-PAGE gel. For the negative control beads were incubated with IgG antibody and cell lysate was applied to IgG antibodies coated beads in a similar manner to SF3B1 antibody-coated beads. Immunoblotting was performed with both anti-RPB1 (664906; BioLegend, San Diego, CA, USA) and anti-SF3B1 antibody [65].

### 4.8. Chromatin Immunoprecipitation (ChIP)

The 1% formaldehyde was used to crosslink HEK293T cells and quenched with 0.125 M glycine. M220 Focused-ultrasonicator (Covaris, Woburn, MA, USA) was then used to fragment crosslinked chromatin. Fragmented chromatin was incubated with antibodies (RNA Pol II antibody (mAb), RNA Pol II antibody (mAb), (39097; Active Motif, Carlsbad, CA, USA) and Dynabeads^®^ Protein A or G (Invitrogen, Carlsbad, CA, USA) to make bead–antibody complexes. After incubation and washing the beads, bound DNA was eluted and purified using Buffer NTB (740595.150; MACHEREY-NAGEL, Düren, Germany) and NucleoSpin Gel and PCR Clean-up (740609.250; MACHEREY-NAGEL, Düren, Germany) [66]. Eluted DNA was used for qPCR and primers are listed in the Supplementary Table S1.

### 4.9. Statistical Analysis

RT-PCR, immunoblotting, and immunoprecipitation analyses were performed in triplicate. Data are presented as mean ± SD (standard deviation of the mean) and the statistical differences among groups were analyzed using the one-way ANOVA tool. Statistical significance was shown as * *p* < 0.05, ** *p* < 0.01, *** *p* < 0.001, and **** *p* < 0.0001.

## Data Availability

The RNA-seq data and m^6^A-seq data are available at the NCBI website under accession number PRJNA1460734.

## Supplementary Materials

The following supporting information can be downloaded at: https://www.mdpi.com/article/10.3390/ijms27125396/s1.

## Abbreviations

The following abbreviations are used in this manuscript:

rMATS	Multivariant analysis of transcript
AS	Alternative splicing
m^6^A	N^6^-methyladenosine
METTL3	Methyltransferase-like 3
