# Supplementary material for: Suppressed SF3B1 Expression Lowers METTL3 Transcription and m6A RNA Expression
Source: Int J Mol Sci. 2026 Jun 15;27(12):5396. doi: 10.3390/ijms27125396 (PMC13300658; doi:10.3390/ijms27125396)

Supplementary Figure S1: IGV Expression peaks of TNTS8, TSR1, RPE65, LRRC57, ARL3 and FRS3 in SF3B1KD vs NS samples.

(a)

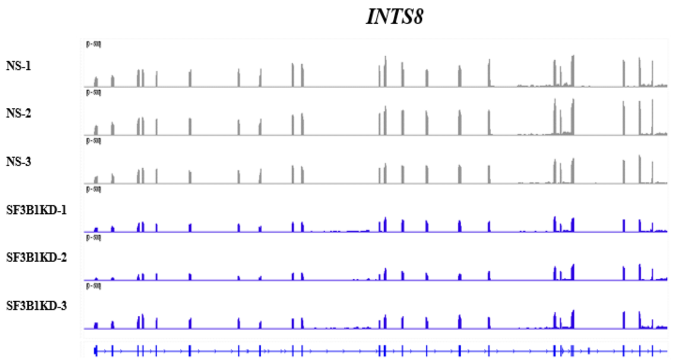

(b)

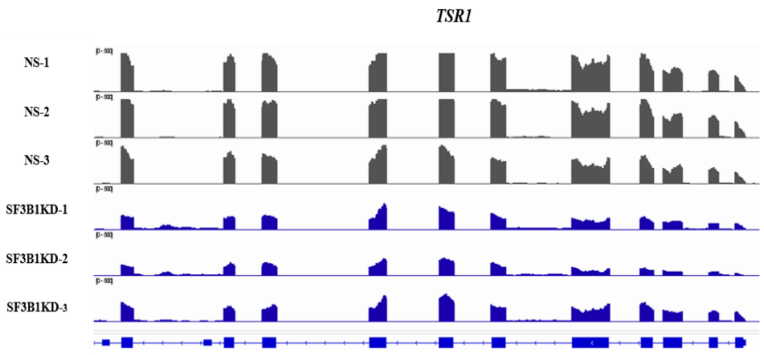

(c)

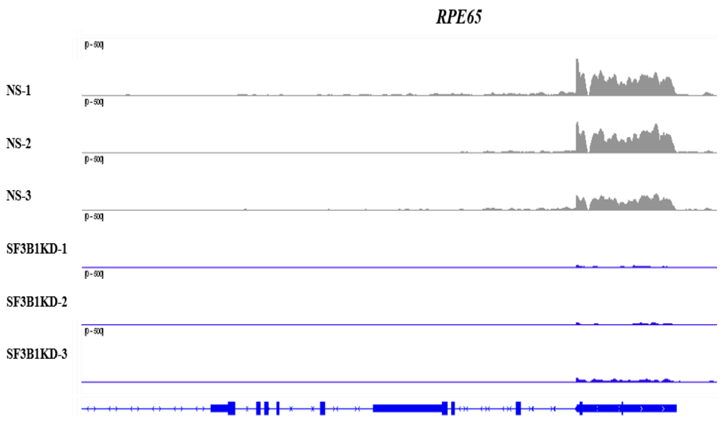

(d)

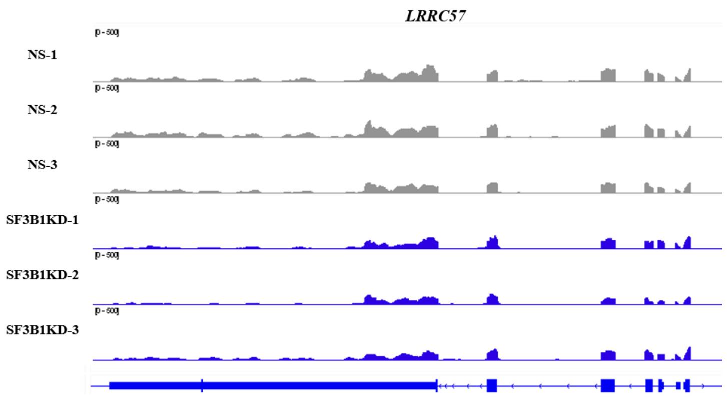

(e)

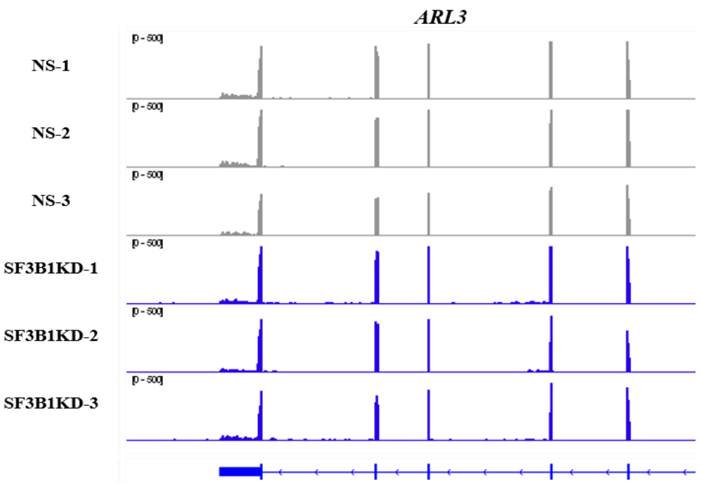

(f)

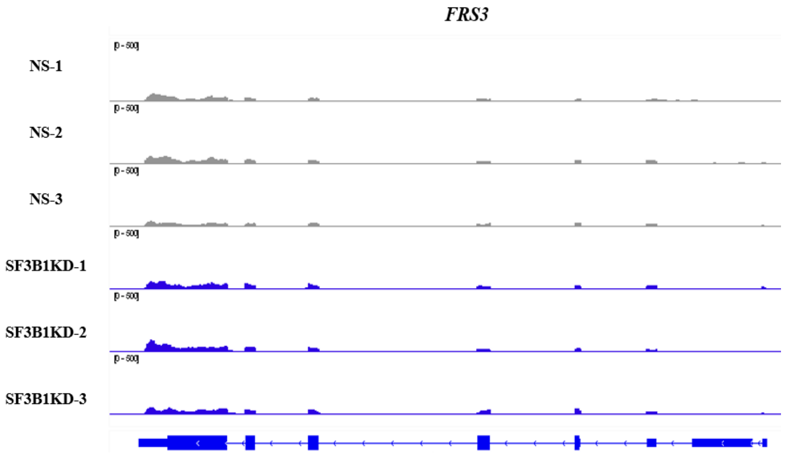

Supplement: Supplementary file 1 [file ijms-27-05396-s001.zip › Supplementary Figure S1.pdf]
